# Editorial: Environmental Exposures and Cardiometabolic Disease

**DOI:** 10.3389/fpubh.2022.895576

**Published:** 2022-06-10

**Authors:** Li Chen, Zengwu Wang, Chunxia Jing, Guang Hao

**Affiliations:** ^1^Department of Medicine, Georgia Prevention Institute, Medical College of Georgia, Augusta University, Augusta, GA, United States; ^2^Division of Prevention and Community Health, National Center for Cardiovascular Disease, National Clinical Research Center of Cardiovascular Disease, State Key Laboratory of Cardiovascular Disease, Fuwai Hospital, Peking Union Medical College & Chinese Academy of Medical Sciences, Beijing, China; ^3^Department of Public Health and Preventive Medicine, School of Medicine, Jinan University, Guangzhou, China

**Keywords:** cardiovascular disease, type 2 diabetes, air pollution, chemical toxic agent, obesity

Cardiometabolic disease, including primarily cardiovascular (CV) disease, diabetes mellitus, and chronic renal failure, is the leading cause of death globally. The elevated risks of cardiometabolic disease are driven mainly by modern lifestyle and environmental changes. There is growing evidence to suggest that exposure to toxic chemicals, noise, and air pollution, are associated with an increased risk of multiple adverse health effects. According to the report from World Health Organization (WHO), it is estimated that, globally, 13.7 million deaths (24.3% of total) and 602 million Disability-Adjusted Life Years (DALYs) (23.1% of total) were attributable to the environmental factors in 2016. Cardiometabolic disease appears to be the most affected one. Of them, up to 4.0 million (29%) deaths and 99 million (16%) DALYs were from CV diseases alone. Identifying and targeting these modifiable risk factors have the greatest potential for reducing the burden of cardiometabolic disease worldwide. Therefore, this Research Topic is to discover new environmental factors, confirm the causal relationships, and explore effective strategies to decrease the risk from harmful exposures.

This Research Topic received four article submissions. Household pesticides are commonly used indoors to control pests and disease vectors in homes and gardens. Pesticides cause not only acute injury but also long-term chronic adverse effects on human health. Using the National Health and Nutrition Examination Survey (NHANES) 1999–2014 data, Chen et al. evaluated whether household pesticide exposure was associated with elevated risks of high blood pressure and if smoking could influence the association between household pesticide exposure and hypertension risk. The result showed that household pesticide exposure was significantly associated with hypertension. In addition, an interaction between smoking status and pesticide exposure on hypertension was reported, indicating that smoking may accentuate the effect of pesticide exposure on blood pressure.

Polycyclic aromatic hydrocarbons (PAHs) are made up of two or more fused benzene rings. These ubiquitous contaminants are formed naturally or by incomplete combustion or through organic matter. PAHs are prevalent environmental contaminants across the world, and chronic CV disease, including heart failure, coronary artery disease, and stroke, has been one of the main global public health concerns. Mallah et al. examined the relationship of polycyclic aromatic hydrocarbon (PAH) exposure with CV diseases in a narrative systematic review. After a comprehensive literature search and summarizing findings from 20 articles, the authors reported that environmental PAH exposure was positively associated with CV disease and well-established CV risk factors in both occupational settings and the general population.

According to the recommendation made by WHO, long-term exposure to an excessive amount of fine Particulate Matter ≤ 2.5 μm (PM2.5) over 10–25 μg/m3 can potentially cause inflammation, impaired coagulation process, and damages to blood vessels, which eventually lead to CV and respiratory diseases as well as cancers. PM2.5-related CV diseases lead to substantial excess medical costs and quality-adjusted life-years (QALYs) loss. However, there is a debate on sex discrepancies for the PM2.5-CV disease associations. Zhang et al. examined the sex differences in associations of ischemic heart disease (IHD) and stroke with long-term PM2.5 exposure in a systematic review and meta-analysis. They identified 25 eligible studies with 3.6 million IHD and 1.3 million stroke cases among 63.7 million participants and reported a significantly stronger association between PM2.5 and risk of IHD in women, compared with men, while the associations of long-term PM2.5 exposure with stroke were significant in both women and men with similar effect magnitudes between sexes.

Children may be sensitive to harmful effects of chemicals, such as PM2.5 exposure, and signs of CV diseases have been found in childhood. The last study conducted by Tong et al. explored the effect of long-term exposure to PM2.5 on childhood obesity in a cohort study. A total of 4,284 children aged 6–8 years at baseline were enrolled from the Chongqing Children Health Cohort in 2014–2015 and were followed up in 2019. In line with the results from previous studies, a higher level of accumulating exposure to PM2.5 was associated with increased childhood obesity indexes, and the effect was more significant for waist-to-height ratio than body mass index. This effect was more pronounced in boys than in girls, which is not consistent with Zhang et al., suggesting that sex differences in the effects of PM2.5 exposure on CV risk are complicated and need to be further studied.

There is consistent evidence of an important role of environmental factors on the effect of environmental exposure on cardiometabolic disease. However, the roles of environmental factors in specific subpopulations are still inconclusive, such as the results of sex differences published in this volume. Additional studies are warranted to confirm these relationships and potential mechanisms, then to build or refine the strategies to eliminate the detrimental health effects from harmful environmental exposure.

## Author Contributions

GH and LC wrote the first draft of this manuscript. ZW and CJ critically reviewed and approved the final paper. All authors contributed to the article and approved the submitted version.

## Conflict of Interest

The authors declare that the research was conducted in the absence of any commercial or financial relationships that could be construed as a potential conflict of interest.

## Publisher's Note

All claims expressed in this article are solely those of the authors and do not necessarily represent those of their affiliated organizations, or those of the publisher, the editors and the reviewers. Any product that may be evaluated in this article, or claim that may be made by its manufacturer, is not guaranteed or endorsed by the publisher.

